# Predisposing and precipitating factors for the development of postoperative delirium in critically ill patients in a university intensive care unit

**DOI:** 10.1590/1518-8345.7113.4233

**Published:** 2024-09-02

**Authors:** Danielle Moreira Marques, Davi da Silveira Barroso Alves, Taís Veronica Cardoso Vernaglia

**Affiliations:** 1 Universidade Federal do Estado do Rio de Janeiro, Rio de Janeiro, RJ, Brazil.; 2 Universidade do Estado do Rio de Janeiro, Hospital Universitário Pedro Ernesto, Rio de Janeiro, RJ, Brazil.; 3 Universidade Federal do Estado do Rio de Janeiro, Departamento de Métodos Quantitativos, Rio de Janeiro, RJ, Brazil.; 4 Universidade Federal do Estado do Rio de Janeiro, Escola de Enfermagem Alfredo Pinto, Rio de Janeiro, RJ, Brazil.

**Keywords:** Delirium, Emergence Delirium, Neurosciense Nursing, Postoperative Care, Measures of Association, Exposure, Risk or Outcome, Critical Care

## Abstract

**Objective:**

to detect the incidence of postoperative delirium in critically ill patients admitted to a surgical intensive care unit and to evaluate the predisposing and precipitating factors associated with postoperative delirium in critically ill patients admitted to a surgical intensive care unit.

**Method:**

this is a prospective cohort study of 157 critically ill surgical patients. Fisher’s exact test and Chi-square test were used for the association between factors and the occurrence of delirium, the Wilcoxon test for numerical variables, and the logistic regression model for the analysis of predisposing and precipitating factors.

**Results:**

the incidence of delirium was 28% (n=44). Age was a significant predisposing factor (p=0.001), followed by the length of surgery (p<0.001), blood transfusion (p=0.043), administration of crystalloids (p=0.008), and anti-inflammatory drugs (p=0.037), which were the precipitating factors identified. The best-adjusted models were: age, length of surgery, non-administration of anti-emetics, use of sufentanil, and blood transfusion.

**Conclusion:**

delirium is a frequent condition in critically ill adults undergoing surgery and the existence of precipitating and predisposing factors is relevant to the outcome, with the anesthetic-surgical procedure as the catalyst event.

## Highlights

**(1)** It elucidates precipitating factors considered unusual in the literature.

**(2)** It corroborates the relational hypothesis of neurotransmitters for the occurrence of delirium.

**(3)** It develops an adjusted model for early identification of susceptible individuals.

**(4)** Evidence that the use of opioids (sufentanil) has a greater impact on delirium.

**(5)** Adds complementarity to the nursing diagnosis of acute confusion.

## Introduction

Delirium consists of an acute and fluctuating dysfunction of the mental state, characterized by a disturbance of attention, consciousness, and cognition, which involves a wide variety of clinical manifestations, with a versatile evolution in the impairment of cognitive functionality^1^. 

Described as a frequent condition in critically ill patients, its incidence can reach 80%, depending on the population studied and the diagnostic criteria used^2^. In patients undergoing anesthetic-surgical procedures, this condition tends to be underreported, reaching 33.1 to 46.2% for those undergoing elective or emergency surgery, respectively^3^, and for every day of delirium, there is a 10% increase in the risk of death^2^.

This condition results in hospital costs, exclusively in the United States, of between 806 and 24,509 dollars and, in intensive care, between 1,529 and 14,462 per individual^4^. In addition to increasing the length of hospital stay and days on mechanical ventilation, delirium represents an undesirable event for patients and their families, since cognitive decline can persist for months or years, impacting quality of life, as well as social and economic impairment^5^. 

Although the predisposing and precipitating factors have not been fully elucidated^5^, it is thought that the establishment of the condition depends on a complex interaction between these factors. Predisposing factors reflect non-changing conditions such as age and comorbidities. Precipitating factors, on the other hand, are usually modifiable and represent insults caused by hospitalization, such as excessive use of technology, pharmacological treatment, and altered sleep-wake patterns^6^. 

Considering that pharmacological strategies are not the first therapeutic option for delirium and are not always effective^2^, it is essential to identify predisposing and precipitating factors. It is also understood that delirium is a nosological entity represented by the nursing diagnosis of acute confusion, defined by reversible disturbances in consciousness, attention, cognition, and perception that develop over a short period^7^. 

Although acute confusion is the result of organic alterations, it is mainly manifested by behavioral alterations recognized by nurses. This professional works to plan and implement interventions aimed at the risk factors identified, as well as preventing and detecting delirium at an early stage, and evaluating the results and progress of the care provided^7^.

Some studies show that there is a greater likelihood of developing delirium during the postoperative period in older individuals^4^. However, there is no consensus regarding the impact of the surgical event on the development of delirium. For example, general anesthesia, in which the isolated use of anesthesia has a higher incidence of cognitive decline when compared to patients who use regional anesthesia^8^ and the prolongation of the anesthetic-surgical procedure^9^ have been shown in the literature to be factors correlated with delirium. In contrast, other authors have considered that predisposing factors related to underlying diseases, such as dementia and heart failure, have a greater impact^10^.

Thus, by understanding the scientific gap in the elucidation of delirium associated with the anesthetic-surgical event and the role of nurses in identifying triggering factors and planning mitigating actions, the objectives are to detect the incidence of postoperative delirium in critically ill patients admitted to a surgical intensive care unit; and to evaluate the predisposing and precipitating factors associated with postoperative delirium in critically ill patients admitted to a surgical intensive care unit.

## Method

### Study type

This is a prospective cohort study with a quantitative approach.

### Scenario

The research took place in a critical surgical unit of a university hospital located in the city of Rio de Janeiro, in the state of Rio de Janeiro, Brazil. The unit consists of seven inpatient beds for male and female patients undergoing major noncardiac surgical procedures. The hospital has an average of 35 admissions per month, with an average length of stay of 2.3 days. The sector provides daily family visits at predetermined times and has adequate infrastructure to offer quality care and safety to people in critical condition.

### Period

The study was carried out from January 5 to July 4, 2022. 

### Population

The study population consisted of 157 eligible patients who agreed to take part in the study and who were fully followed up during the data collection period. For this study, there was no loss to follow-up.

### Selection criteria

Patients in the immediate postoperative period in a non-cardiac surgical critical care unit, over 18 years of age, were included. Patients undergoing intracranial surgery were considered as an exclusion criterion, given that this surgical modality can represent a confounding bias for neurological assessment.

### Participants

Considering the average of 35 hospitalizations per month in the study setting and the determined follow-up time, an estimate of 210 hospitalizations over six months was obtained. The sample size was defined using a 95% confidence interval (CI) and a maximum margin of error for the proportion of 5%, resulting in 137 participants. It is worth noting that no sampling process was carried out to select the participants, but the sample calculation was used as a basis for the study’s follow-up period.

### Instruments used to collect information

A literature review was carried out on the risk factors for the development of postoperative delirium (POD) in order to define the variables that made up the data collection instrument, based on the following axes: sociodemographic data, health history, intraoperative occurrences, and post-surgical follow-up in intensive care.

The occurrence of delirium was verified using the Confusion Assessment Method for the Intensive Care Unit (CAM-ICU) - a validated method for identifying delirium. This tool, which was developed in 2001, requires the use of the Richmond Agitation and Sedation Scale (RASS) and is composed of the following items: acute onset or fluctuating course of the condition, attention disturbance, disorganized thinking, and altered level of consciousness^12^. The instrument has been validated for Portuguese and has high sensitivity and specificity. It is used at the bedside and requires minimal training^13^. 

### Data collection

Data collection was carried out by thirteen nurses working in the study setting during the data collection period. These are professionals who have worked in intensive care for more than two years and who were trained by the main researcher responsible for the study to evaluate and apply the data collection instruments. The training consisted of a presentation of the data collection instruments, clarification of the ethical aspects, and the steps for applying the CAM-ICU method.

Participants were approached immediately on admission and were followed up until discharge from the critical surgical unit. Sociodemographic and clinical characteristics were obtained from medical records and interviews. Postoperative delirium was considered to be acute confusion identified within one week of the anesthetic-surgical procedure^14^. Data collection was planned to take place both during the day and at night, considering that acute confusional states tend to fluctuate and manifest more frequently at night^2^.

### Data processing and analysis

The data obtained was categorized into predisposing factors (age, gender, history of previous surgery, hypertension, diabetes mellitus, chronic renal failure, dementia, stroke, psychiatric involvement, alcoholism, visual or hearing impairment), and precipitating factors (use of illicit drugs, use of illicit drugs, antihypertensives, antipsychotics, antibiotic therapy, benzodiazepines, and opioids during the pre-surgical period, emergency or elective nature and length of surgery, use of crystalloids, colloids, antibiotics, anticholinergics, analgesics, anti-inflammatories, antiemetics, anesthetic agents and blood transfusions).

The variables were then tabulated using the Microsoft^®^ Excel computer program and then analyzed using the R software (version 4.2). The association between the selected factors and the occurrence of delirium was analyzed using Fisher’s exact test and the Chi-square test, considering a significance level of 5%, while numerical variables were compared using the Wilcoxon test.

A logistic regression model was used to assess the effect of each of the factors analyzed on the chances of delirium occurring. The modeling was carried out in three stages. In the first stage, a simple regression model was adjusted for each factor analyzed; in the second stage, a multiple model was adjusted with the variables that were significant at 5% in the simple regression, and those that lost significance were removed. In the third stage, the variables relating to each of the predisposing factors, precipitants, anesthetic agents, and pharmacological classes with significance at 20% in the simple regression were evaluated in multiple models, with only those with significance of 5% being kept. The modeling method adopted in this study advocates a purposive selection model in which, initially, a multiple model is adjusted including the independent variables that obtained significance of up to 25% in the simple regression. Next, the non-significant variables at 5% are removed one at a time and, after considering all the variables, the return effect of each variable removed from the initial model is tested in order to check whether the previously removed independent variable can adopt a differential behavior in the presence of other variables^15^.

### Ethical aspects

The project was approved by the Research Ethics Committee under opinion no. 5.051.627 and was conducted on a voluntary and confidential basis. Participants were approached before the anesthetic-surgical procedure, in a preserved cognitive state, and informed about the objectives, methodology, potential risks arising from their participation, and the possibility of leaving the study at any time.

The risks inherent in this research were considered low, as the data collected was part of the unit’s routine and the research team are professionals who work in the sector. In order to mitigate the risk related to the misuse of participants’ information, training was offered to the research team.

## Results

A total of 236 patients were seen in the sector during the period, of which 71 were excluded due to the exclusion criteria, and eight, even in a preserved cognitive state, refused to take part in the study, totaling 157 participants. As shown in Table 1, the participants had a median age of 63 years, ranging from 23 to 88 years, a predominance of females (n=84; 54%) and hypertensive patients (n=104; 67%). The following chronic diseases were also found: *Diabetes Mellitus* (n=57; 37%), alcoholism (n=28; 18%), psychiatric illness (n=17; 11%), chronic renal failure (n=11; 7.1%), stroke (n=10; 6.4%) and dementia (n=3; 1.9%). There were reports of previous use of antihypertensive drugs (n=96; 61.1%), antipsychotics (n=21; 13.4%), antibiotics (n=10; 6.7%), opioids (n=11; 12.6%), benzodiazepines (n=12; 13.8%) and illicit drugs (n=3; 1.9%).

**Table 1:** Precipitating and predisposing factors for delirium in adult critically ill patients at a university hospital. Rio de Janeiro, RJ, Brazil, 2023

Total			Delirium				Simple Regression			
Variables	N	n = 157*	n	No (n = 113)*	Yes (n = 44)*	p-value	n	OR^†^	CI95% ^‡^	p-value
**Sex**	157		157			0,365^§^	157			
Female		84 (54%)		63 (75%)	21 (25%)			-	-	
Male		73 (46%)		50 (68%)	23 (32%)			1.38	0.69, 2.79	0.4
**Age**	157	63 (50, 71)^||^	157	60 (48, 69)^||^	70 (60, 74)^||^	0,001^¶^	157	1.04	1.01, 1.07	0.005
**Time of surgery**	143	240 (158, 302)^||^	143	200 (148, 280)^||^	270 (218, 394)^||^	<0,001^¶^	143	1.00	1.00, 1.01	0.001
**Hypertension**	156		156			0,166^§^	156			
No		52 (33%)		41 (79%)	11 (21%)			-	-	
Yes		104 (67%)		71 (68%)	33 (32%)			1.73	0.81, 3.92	0.2
***Diabetes Mellitus***	156		156			0,147^§^	156			
No		99 (63%)		75 (76%)	24 (24%)			-	-	
Yes		57 (37%)		37 (65%)	20 (35%)			1.69	0.83, 3.45	0.15
**Dementia**	156		156			0,192**	156			
No		153 (98%)		111 (73%)	42 (27%)			-	-	
Yes		3 (1.9%)		1 (33%)	2 (67%)			5.29	0.49, 116	0.2
**Alcoholism**	154		154			0,138^§^	154			
No		126 (82%)		94 (75%)	32 (25%)			-	-	
Yes		28 (18%)		17 (61%)	11 (39%)			1.90	0.79, 4.45	0.14
**Visual impairment**	156		156			0,116^§^	156			
No		83 (53%)		64 (77%)	19 (23%)			-	-	
Yes		73 (47%)		48 (66%)	25 (34%)			1.75	0.87, 3.58	0.12
**Hearing impairment**	155		155			0,223**	155			
No		141 (91%)		103 (73%)	38 (27%)			-	-	
Yes		14 (9.0%)		8 (57%)	6 (43%)			2.03	0.63, 6.23	0.2
**Intraoperative transfusion**	153		153			0,043**	153			
No		136 (89%)		101 (74%)	35 (26%)			-	-	
Yes		17 (11%)		8 (47%)	9 (53%)			3.25	1.16, 9.29	0.025
**Intraoperative crystalloid**	157		157			0,008^§^	157			
No		73 (46%)		60 (82%)	13 (18%)			-	-	
Yes		84 (54%)		53 (63%)	31 (37%)			2.70	1.30, 5.84	0.009
**Intraoperative colloid**	157		157			0,252**	157			
No		140 (89%)		103 (74%)	37 (26%)			-	-	
Yes		17 (11%)		10 (59%)	7 (41%)			1.95	0.66, 5.45	0.2
**Intraoperative antibiotics**	157		157			1,000**	157			
No		147 (94%)		106 (72%)	41 (28%)			-	-	
Sim		10 (6.4%)		7 (70%)	3 (30%)			1.11	0.23, 4.20	0.9
**Intraoperative anticholinergic**	153		153			0,343^§^	153			
Yes		28 (18%)		22 (79%)	6 (21%)			-	-	
No		125 (82%)		87 (70%)	38 (30%)			1.60	0.63, 4.63	0.3
**Intraoperative anti-inflammatory**	153		153			0,037^§^	153			
Yes		30 (20%)		26 (87%)	4 (13%)			-	-	
No		123 (80%)		83 (67%)	40 (33%)			3.13	1.13, 11.1	0.045
**Intraoperative antiemetic**	152		152			0,129^§^	152			
Yes		97 (64%)		73 (75%)	24 (25%)			-	-	
No		55 (36%)		35 (64%)	20 (36%)			1.74	0.85, 3.57	0.13
**Intraoperative lidocaine**	152		152			0,139^§^	152			
No		66 (43%)		51 (77%)	15 (23%)			-	-	
Yes		86 (57%)		57 (66%)	29 (34%)			1.73	0.84, 3.65	0.14
**Intraoperative sufentanil**	152		152			0,117**	152			
No		138 (91%)		101 (73%)	37 (27%)			-	-	
Yes		14 (9.2%)		7 (50%)	7 (50%)			2.73	0.88, 8.49	0.077
**Intraoperative midazolam**	152		152			0,100^§^	152			
Yes		117 (77%)		87 (74%)	30 (26%)			-	-	
No		35 (23%)		21 (60%)	14 (40%)			1.93	0.86, 4.26	0.10
**Intraoperative propofol**	152		152			0,230^§^	152			
Yes		120 (79%)		88 (73%)	32 (27%)			-	-	
No		32 (21%)		20 (62%)	12 (38%)			1.65	0.71, 3.73	0.2
**Intraoperative sevoflurane**	153		153			0,196^§^	153			
No		112 (73%)		83 (74%)	29 (26%)			-	-	
Yes		41 (27%)		26 (63%)	15 (37%)			1.65	0.76, 3.53	0.2

^†^OR = Odds Ratio; ^‡^CI = Confidence Interval; ^§^Pearson’s Chi-square test; ^||^Median (IQR: Interquartile Range); ^¶^Wilcoxon test; **Fisher’s exact test

With regard to the anesthetic-surgical procedure, the median intraoperative time was 240 minutes, the shortest being 20 minutes and the longest 705 minutes. There was a predominance of intravenous crystalloids (n=84; 54%), as opposed to colloid infusion (n=17; 11%), and there was a need for transfusion during surgery for 17 participants (11%). The following drugs were administered during surgery: analgesics (n=119; 78%), antiemetics (n=97; 64%), anti-inflammatories (n=30; 20%), and anticholinergics (n=28; 18%).

The participants had a history of previous surgeries (n=122; 77.7%) on an elective basis (n=145; 93.5%) and underwent the following specialties: general surgery (n=51; 32.6%), thoracic (n=39; 25%), vascular (n=28; 17.9%), urological (n=6; 3.8%), gynecological (n=5; 3.2%); orthopedic (n=3; 1.9%) head and neck (n=1; 0.6%), neurosurgery (n=1; 0.6%). A variety of anesthetic techniques were used: general anesthesia (n=88; 56%), inhalation (n=35; 22.2%), general combined with epidural (n=27; 17.2%), local (n=3; 1.9%), epidural (n=2; 1.2%), subarachnoid block (n=1; 0.6%) or sedation alone (n=1; 0.6%).

The incidence of delirium in the study population was 28% (n=44). Older age was a statistically significant predisposing factor (p=0.001) and this was shown by the significant difference between the median age for delirious participants (70 years) compared to non-delirious participants (60 years). With regard to the precipitating factors assessed during the intraoperative period, there was significance in terms of the length of surgery (p<0.001), the use of blood transfusion (p=0.043), the administration of crystalloids (p=0.008) and anti-inflammatory drugs (p=0.037) for the delirium outcome.

Multiple models were adjusted with the variables that were significant at 5%, and those that lost significance were removed, resulting in a model with age and surgery time. From this, significant variables at 20% were added, resulting in an adjusted model made up of the following variables: age, length of surgery, administration of antiemetics, use of sufentanil, and blood transfusion during the intraoperative period, as shown in Table 2.

**Table 2:** Multivariate logistic regression model of precipitating and predisposing factors for delirium in adult critically ill patients at a university hospital. Rio de Janeiro, RJ, Brazil, 2023

	Initial Model			Final Model		
Features	OR*	CI95%^†^	p-value	OR*	CI95%^†^	p-value
**Age**	1.038	1.011, 1.068	0.008	1.037	1.010, 1.069	0.010
**Time of surgery**	1.005	1.002, 1.008	0.001	1.004	1.001, 1.008	0.005
**Intraoperative antiemetic**						
Yes				-	-	
No				2.895	1.196, 7.229	0.020
**Intraoperative sufentanil**						
No				-	-	
Yes				5.748	1.488, 23.66	0.012
**Intraoperative transfusion**						
No				-	-	
Yes				3.814	1.064, 14.29	0.041

*OR = Odds Ratio; ^†^CI= Confidence Interval

## Discussion

The incidence of postoperative delirium (28%) is moderately higher than that reported in the literature, which indicates 24% for elderly patients undergoing non-cardiac surgery^16^ and 26.6% for elderly patients undergoing orthopedic procedures^8^. 

This study found an increase in age (OR: 1.037). Although the literature points to the impact of health history on the development of delirium^10^, this association was not found in this study. However, it was found that age is a significant predisposing factor and that each year of life increases the risk of delirium by 3.7%. This is due to the apoptosis of neurons, a decrease in cerebral blood flow, and changes in the neurotransmitter system^4^.

The duration of the anesthetic-surgical procedure proved to be significant, with each minute of surgery increasing the chance of delirium by 0.4%. This is in line with other authors, who highlight the higher incidence of delirium in patients undergoing thoracic surgeries lasting more than 80 minutes^17^, justifying the recommendation that head and neck surgeries should be performed by two surgeons, with the aim of shortening the duration of the procedure^18^.

The anti-inflammatory drugs identified during surgery consist of selective cyclooxygenase-2 inhibitors (parecoxib) and non-selective cyclooxygenase-2 inhibitors (ketoprofen) and were significantly associated with POD. Although the impact of the use of this pharmacological class has not been elucidated, it has been suggested that cytokines, understood as a group of peptide cell mediators produced from an inflammatory response, may play a role in triggering delirium due to the increased permeability of the blood-brain barrier^19^. 

The variable with the greatest impact was the intraoperative use of sufentanil, an opioid with five to 10 times greater potency when compared to fentanyl^20^, resulting in a 474% increase in the chance of delirium occurring (OR: 5.748). This finding is corroborated by another prospective cohort study of patients undergoing elective radical prostatectomy^9^. One challenge regarding the use of opioids and the development of delirium is determining the effective dose. The literature highlights that pain is a high-risk factor and increases the chance of delirium (9.85 times), which points to the need for a systematized approach to the assessment and management of perioperative pain^21^. 

This study also reveals that 53% of those who received a blood transfusion during the intraoperative period had delirium which reflects a 281% increase in the chance of delirium occurring (OR: 3.814). Anemia is one of the most predictive precipitating factors^21^ and intraoperative bleeding is considered a risk factor for delirium, especially when it exceeds a loss of 400 ml^8^.

During the intraoperative period, volume replacement aims to promote fluid or hemodynamic maintenance^22^. However, this therapy is usually carried out through the administration of crystalloids, either through the use of the saline solution or Ringer’s solution, which leads to a drop in plasma colloid osmotic pressure, resulting in the loss of fluids into the third space. In this context, a study on the prevention of hypotension during spinal anesthesia during cesarean section showed that fewer patients had hypotension in the colloid group compared to the crystalloid group^22^. 

The literature points to causes secondary to the need for volume replacement as precipitating factors for delirium, such as the association between delirium and hypotension during the anesthetic-surgical procedure^23^ and dehydration during the postoperative period^21^. However, this study differs in that it evaluates the nature of water replacement, highlighting the use of crystalloids as a significant event when compared to the use of colloids. 

Although pharmacological management of delirium has not proven to be an effective strategy, some studies on the prevention or treatment of post-surgical delirium have focused on the possibility of ondansetron being a promising drug. Delirium is an important manifestation of serotonergic syndrome and ondansetron is a serotonin antagonist specific for the 5-HT3 receptor and is routinely used during the postoperative period to control nausea and vomiting^24^.

Although this study was not intended to present ondansetron as a pharmacological strategy for the management of delirium, it should be noted that not using this drug during the intraoperative period was associated with a 198% increase (OR: 2.895) in the chance of delirium occurring. Therefore, this finding is relevant as it provides support for the relational hypothesis of the neurotransmitters that trigger delirium, especially serotonin.

However, although this study has shown an association between predisposing and precipitating factors and delirium, some limitations should be made clear, such as the fact that the doses of the drugs evaluated were not individualized, as well as the fact that the study was conducted in a highly complex university hospital that does not provide emergency care, raising the possibility that the population studied has different surgical characteristics, and that the findings may not be generalizable. 

In addition, it was impossible to obtain some of the study variables for all participants, which were tabulated as missing data due to incomplete information in medical records. Others could not be assessed by this study, such as previous nutritional status and surgical risk classification according to the American Society of Anesthesiology (ASA), data considered relevant to the occurrence of delirium, but which was absent from the participants’ medical records.

It should be noted that although there is a correlation between precipitating and predisposing factors and postoperative delirium, it is understood that this does not consolidate causality and that the associated factors may be related to an underlying cause that has not yet been elucidated in the literature.

In this case, it is hoped that the identification of risk factors carried out in this study will contribute to the scientific direction of the pathophysiological elucidation of delirium and also support future publications aimed at validating interventions aimed at reducing the risk of POD.

## Conclusion

It was found that delirium is a frequent occurrence during the postoperative period in critically ill adults. Given that intensive care is a favorable environment for delirium, either due to the underlying situation of confinement or the unfavorable organic condition of its patients, and with the anesthetic-surgical procedure as the catalyst event, the identification of factors associated with delirium is postulated as a mitigating strategy.

Although some predisposing factors for delirium are well established in the literature, this study differs in that it establishes precipitating factors related to the intraoperative period, such as surgical time, the use of sufentanil, the use of blood transfusion, the infusion of anesthetic agents, as well as the non-administration of ondansetron, a serotonin antagonist, and therefore corroborates the relational hypothesis of neurotransmitters for the development of delirium.

This highlights the role of nurses in surgical intensive care both in identifying delirium through the use of validated instruments and in detecting associated factors, especially in the elderly and patients who have undergone prolonged surgery, who are more likely to develop this dysfunction. The aim is to provide support for the systematization of perioperative nursing care, insofar as this work adds complementarity to the nursing diagnosis called acute confusion.

This study is expected to make a vast social contribution insofar as it addresses a common disease among the elderly in a global scenario of population aging. It is also believed to reduce socio-economic costs as a result of improving the quality of health care, as well as increasing life expectancy and encouraging the implementation of innovative care technologies.

## References

[1] American Psychiatric Association (2013). Diagnostic and Statistical Manual of Mental Disorders.

[2] Itaborahy RS, Lima LS (2023). Delirium na Unidade de Terapia Intensiva. REAMed.

[3] Cherak SJ, Soo A, Brown KN, Ely EW, Stelfox HT, Fiest KM (2020). Development and validation of delirium prediction model for critically ill adults parameterized to ICU admission acuity. PLoS One.

[4] Kinchin I, Mitchell E, Agar M, Trépel D (2021). The economic cost of delirium: A systematic review and quality assessment. Alzheimers Dement.

[5] Esmaeeli S, Franco-Garcia E, Akeju O, Heng M, Zhou C, Azocar RJ (2022). Association of preoperative frailty with postoperative delirium in elderly orthopedic trauma patients. Aging Clin Exp Res.

[6] Pinheiro FG, Santos ES, Barreto ID, Weiss C, Oliveira JC, Vaez AC (2022). Prevalence and risk factors associated with delirium at a critical care unit. Acta Paul Enferm.

[7] Gomes A, Rosinhas A, Ramos S, Sampaio F (2023). Autonomous nursing interventions to prevent acute confusion: integrative literature review. Rev Port Enferm Saúde Mental.

[8] Wang H, Zhang L, Zhang Z, Li Y, Luo Q, Yuan S (2020). Perioperative Sleep Disturbances and Postoperative Delirium in Adult Patients: A Systematic Review and Meta-Analysis of Clinical Trials. Front Psychiatry.

[9] Stuff K, Kainz E, Kahl U, Pinnschmidt H, Beck S, von Breunig F (2022). Effect of sedative premedication with oral midazolam on postanesthesia care unit delirium in older adults: a secondary analysis following an uncontrolled before-after design. Perioper Med (Lond).

[10] Marquetand J, Gehrke S, Bode L, Fuchs S, Hildenbrand F, Ernst J (2022). Delirium in trauma patients: a 1-year prospective cohort study of 2026 patients. Eur J Trauma Emerg Surg.

[11] Barbetta PA (2012). Estatística Aplicada às Ciências Sociais.

[12] Ely EW, Margolin R, Francis J, May L, Truman B, Dittus R (2001). Evaluation of delirium in critically ill patients: validation of the Confusion Assessment Method for the Intensive Care Unit (CAM-ICU). Crit Care Med.

[13] Mariz JA, Pires O, Lopes A, Bessa J, Correia L, Morgado P (2022). The Confusion Assessment Method for Intensive Care Unit: A Large Cohort Validation Study in the Emergency Department of a Tertiary Hospital. RSPMI.

[14] Evered L, Silbert B, Knopman DS, Scott DA, DeKosky ST, Rasmussen LS (2018). Recommendations for the nomenclature of cognitive change associated with anaesthesia and surgery-2018. Br J Anaesth.

[15] Silveira MBG, Barbosa NFM, Peixoto APB, Xavier EFM, Xavier SFA (2021). Application of logistic regression in the analysis of risk factor associated with arterial hypertension. RSD.

[16] Ho MH, Nealon J, Igwe E, Traynor V, Chang HR, Chen KH (2021). Postoperative Delirium in Older Patients: A Systematic Review of Assessment and Incidence of Postoperative Delirium. Worldviews Evid Based Nurs.

[17] Tong C, Huang C, Wu J, Xu M, Cao H (2020). The Prevalence and Impact of Undiagnosed Mild Cognitive Impairment in Elderly Patients Undergoing Thoracic Surgery: A Prospective Cohort Study. J Cardiothorac Vasc Anesth.

[18] Kolk A, Schwarzer C, Wolff KD, Grill F, Weingart J (2022). Factors Associated With Postoperative Delirium in Patients Undergoing Complex Head and Neck Flap Surgery. J Oral Maxillofac Surg.

[19] Khan BA, Perkins AJ, Prasad NK, Shekhar A, Campbell NL, Gao S (2020). Biomarkers of Delirium Duration and Delirium Severity in the ICU. Crit Care Med.

[20] Matioli KBB, Moraes IM, Sousa TV, Pereira MC, Silva RM, Sá ES (2021). Delirium: prevalence and factors associated with postoperative period of cardiovascular surgery in the elderly. Rev Baiana Enferm.

[21] Carvalho LAC, Correia MDL, Ferreira RC, Botelho ML, Ribeiro E, Duran ECM (2022). Accuracy of delirium risk factors in adult intensive care unit patients. Rev Esc Enferm USP.

[22] Chooi C, Cox JJ, Lumb RS, Middleton P, Chemali M, Emmett RS (2020). Techniques for preventing hypotension during spinal anaesthesia for caesarean section. Cochrane Database Sys Rev.

[23] Maheshwari K, Ahuja S, Khanna AK, Mao G, Perez-Protto S, Farag E (2020). Association Between Perioperative Hypotension and Delirium in PostoperatiQve Critically Ill Patients: A Retrospective Cohort Analysis. Anesth Analg.

[24] Haque N, Naqvi RM, Dasgupta M (2019). Efficacy of Ondansetron in the Prevention or Treatment of Post-operative Delirium-a Systematic Review. Can Geriatr J.

