# Supplementary material for: Predisposing and precipitating factors for the development of postoperative delirium in critically ill patients in a university intensive care unit
Source: Rev Lat Am Enfermagem. 2024 Sep 2;32:e4233. doi: 10.1590/1518-8345.7113.4233 (PMC11386484; doi:10.1590/1518-8345.7113.4233)
Supplement: Translation

# Translation: Fatores predisponentes e precipitantes para o desenvolvimento de *delirium* pós-operatório em pacientes críticos de uma unidade intensiva universitária

**DOI:** 10.1590/1518-8345.7113.4234

**Authors:** Danielle Moreira Marques, Davi da Silveira Barroso Alves, Taís Veronica Cardoso Vernaglia

**Affiliations:** 1 Universidade Federal do Estado do Rio de Janeiro, Rio de Janeiro, RJ, Brasil.; 2 Universidade do Estado do Rio de Janeiro, Hospital Universitário Pedro Ernesto, Rio de Janeiro, RJ, Brasil.; 3 Universidade Federal do Estado do Rio de Janeiro, Departamento de Métodos Quantitativos, Rio de Janeiro, RJ, Brasil.; 4 Universidade Federal do Estado do Rio de Janeiro, Escola de Enfermagem Alfredo Pinto, Rio de Janeiro, RJ, Brasil.

**Keywords:** Delirium, Delírio do Despertar, Enfermagem em Neurociência, Cuidados Pós-Operatórios, Medidas de Associação, Exposição, Risco ou Desfecho, Cuidados Críticos

## Abstract

**Objetivo:**

detectar a incidência de *delirium* pós-operatório em pacientes críticos internados em uma unidade de terapia intensiva cirúrgica e avaliar os fatores predisponentes e precipitantes associados ao *delirium* pós-operatório em pacientes críticos internados em uma unidade de terapia intensiva cirúrgica.

**Método:**

trata-se de um estudo de coorte prospectivo com 157 pacientes críticos cirúrgicos. Utilizou-se teste Exato de Fisher e qui-quadrado para a associação entre os fatores e ocorrência de *delirium*, teste de Wilcoxon para as variáveis numéricas e o modelo de Regressão Logística para a análise dos fatores predisponentes e precipitantes.

**Resultados:**

a incidência de *delirium* foi 28% (n=44). O fator idade apresentou-se predisponente significativo (p=0,001), seguido do tempo de cirurgia (p<0,001), a transfusão sanguínea (p=0,043), a administração de cristaloides (p=0,008) e anti-inflamatórios (p=0,037), estes foram os fatores precipitantes identificados. Obteve-se como melhor modelo ajustado: idade, tempo de cirurgia, a não administração de antieméticos, o uso de sufentanil e a transfusão sanguínea.

**Conclusão:**

o *delirium* consiste em acometimento frequente para adultos críticos cirúrgicos e a existência de fatores precipitantes e predisponentes tornam-se relevantes para o desfecho, tendo-se o procedimento anestésico-cirúrgico como evento catalisador.

## Destaques

**(1)** Elucida fatores precipitantes considerados insólitos na literatura.

**(2)** Corrobora a hipótese relacional de neurotransmissores para ocorrência de *delirium*.

**(3)** Desenvolve um modelo ajustado para identificação precoce de indivíduos suscetíveis.

**(4)** Evidencia que o uso de opioide (sufentanil) acarreta maior impacto para o *delirium*.

**(5)** Agrega complementaridade ao diagnóstico de Enfermagem denominado confusão aguda.

## Introdução

O *delirium* consiste em uma disfunção aguda e flutuante do estado mental, caracterizada por um distúrbio da atenção, consciência e cognição, o qual envolve uma ampla variedade de manifestações clínicas, com evolução versátil no comprometimento da funcionalidade cognitiva^1^. 

Descrito como um acometimento frequente em pacientes críticos, sua incidência pode chegar a 80%, a depender da população estudada e dos critérios diagnósticos utilizados^2^. Em pacientes submetidos aos procedimentos anestésico-cirúrgicos, essa condição tende a ser subnotificada, atingindo de 33,1 a 46,2% para aqueles submetidos às cirurgias eletivas ou de emergência, respectivamente^3^, e, para cada dia em *delirium*, há um incremento de 10% no risco de morte^2^.

Tal condição implica em gasto hospitalar, exclusivamente nos Estados Unidos, que varia entre 806 e 24.509 dólares e, na terapia intensiva, entre 1.529 e 14.462 por indivíduo^4^. Além de aumentar o tempo de permanência hospitalar e nos dias em ventilação mecânica, o *delirium* representa um evento indesejável aos pacientes e familiares, uma vez que o declínio cognitivo pode persistir por meses ou anos, acarretando um impacto na qualidade de vida, bem como o comprometimento social e econômico^5^. 

Ainda que os fatores predisponentes e precipitantes não estejam elucidados por completo^5^, suscita-se que o estabelecimento do quadro dependa de uma complexa interação entre tais fatores. Os fatores predisponentes refletem as condições não mutáveis como a idade e as comorbidades. Já os fatores precipitantes costumam ser passíveis de modificação e representam insultos ocasionados pela hospitalização como emprego excessivo de tecnologia, tratamento farmacológico e alteração de sono-vigília^6^. 

Ao considerar que as estratégias farmacológicas não consistem em primeira opção terapêutica para o *delirium* e nem sempre se mostram eficazes^2^, torna-se imprescindível identificar os fatores predisponentes e precipitantes. Além disso, entende-se que o *delirium* consiste em uma entidade nosológica representada pelo diagnóstico de Enfermagem denominado *confusão aguda*, definido por perturbações reversíveis de consciência, atenção, cognição e percepção que se desenvolvem em um curto período^7^. 

Embora os quadros confusionais agudos decorram de alterações orgânicas, estes manifestam-se, sobretudo, por alterações comportamentais reconhecidas pelo enfermeiro. Este profissional atua no planejamento e na implementação de intervenções direcionadas aos fatores de risco identificados, bem como na prevenção e na detecção precoce do *delirium*, avaliação dos resultados e evolução dos cuidados determinados^7^.

Alguns estudos revelam que há maior probabilidade para o desenvolvimento de *delirium* durante o período pós-operatório de indivíduos mais idosos^4^. No entanto, não há consenso em relação ao impacto do evento cirúrgico para o desenvolvimento *delirium*. Por exemplo, destaca-se a anestesia geral, na qual o emprego isolado denota maior incidência de declínio cognitivo quando comparado aos pacientes que a utilizam em caráter regional^8^ e o prolongamento do procedimento anestésico-cirúrgico^9^, os quais foram evidenciados na literatura como fatores correlacionados ao *delirium*. Em contraste, outros autores consideraram que os fatores predisponentes relacionados às doenças de base, como a demência e a insuficiência cardíaca, denotam fatores de maior impacto^10^.

Desse modo, ao compreender a lacuna científica sobre a elucidação do *delirium* associado ao evento anestésico-cirúrgico e o papel do enfermeiro para a identificação de fatores desencadeadores e planejamento de ações mitigadoras, têm-se por objetivos detectar a incidência de *delirium* pós-operatório em pacientes críticos internados em uma unidade de terapia intensiva cirúrgica; e avaliar os fatores predisponentes e precipitantes associados ao *delirium* pós-operatório em pacientes críticos internados em uma unidade de terapia intensiva cirúrgica.

## Método

### Tipo de estudo

Trata-se de um estudo de coorte prospectivo e de abordagem quantitativa.

### Cenário

A pesquisa ocorreu em uma unidade crítico cirúrgica de um hospital universitário situado no município do Rio de Janeiro, no estado do Rio de Janeiro, Brasil. A unidade é composta por sete leitos de internação destinados aos indivíduos masculinos e femininos, submetidos aos procedimentos cirúrgicos não cardiológicos de grande porte. Este hospital realiza, em média, 35 internações mensais, com tempo médio de permanência que consiste em 2,3 dias. O setor assegura a visita familiar diária em horários predeterminados, e possui infraestrutura adequada à oferta de assistência de qualidade e segurança às pessoas em estado crítico.

### Período

O estudo foi realizado no período de cinco de janeiro a quatro de julho de 2022. 

### População

A população do estudo foi composta por 157 pacientes elegíveis que aceitaram participar da pesquisa, e que foram acompanhados integralmente durante o período de coleta de dados. Para este estudo, não houve perda de seguimento.

### Critérios de seleção

Foram incluídos os pacientes durante o período pós-operatório imediato em unidade crítico cirúrgica não cardiológica, maiores de 18 anos e foram considerados como critério de exclusão os pacientes submetidos à cirurgia intracraniana, compreendendo que esta modalidade cirúrgica pode representar um viés confusional para a avaliação neurológica.

### Participantes

Considerou-se a média de 35 internações mensais no cenário de estudo e o tempo de seguimento determinado, obtendo-se uma estimativa em 210 internações em seis meses. Para a definição de tamanho amostral, adotou-se o Intervalo de Confiança (IC) de 95% e margem de erro máxima para proporção de 5%, obtendo-se 137 participantes^11^. Cabe ressaltar que não foi realizado o processo de amostragem para a seleção dos participantes, utilizando-se o cálculo amostral como subsídio para fundamentar o período de seguimento do estudo.

### Instrumentos utilizados para a coleta das informações

Realizou-se uma revisão da literatura sobre os fatores de risco para o desenvolvimento de *Delirium* Pós-Operatório (DPO) com fins de definição das variáveis que compuseram o instrumento de coleta de dados, com base nos seguintes eixos: dados sociodemográficos, histórico de saúde, ocorrências intraoperatórias e acompanhamento pós-cirúrgico na terapia intensiva.

A ocorrência do *delirium* foi constatada pelo emprego do *Confusion Assessment Method for the Intensive Care Unit* (CAM-ICU) - método validado para a identificação de *delirium*. Esta ferramenta, que foi elaborada em 2001, requer a utilização da Escala de Agitação e Sedação de Richmond (RASS), e é composta pela avaliação dos itens: início agudo ou curso flutuante do quadro, distúrbio da atenção, pensamento desorganizado e alteração do nível de consciência^12^. O instrumento é validado para a língua portuguesa e possui alta sensibilidade e especificidade. Seu emprego se dá à beira-leito e exige treinamento mínimo^13^. 

### Coleta de dados

A coleta de dados foi realizada por treze enfermeiros que atuavam no cenário de estudo, durante o período da coleta de dados. São profissionais com tempo de atuação nas terapias intensivas superior a dois anos, e que receberam treinamento por parte da pesquisadora principal responsável pelo estudo para a avaliação e a aplicação dos instrumentos de coleta de dados. O treinamento consistiu na apresentação dos instrumentos para a coleta das informações, esclarecimento sobre os aspectos éticos e as etapas para a aplicação do método CAM-ICU.

Os participantes eram abordados imediatamente à admissão e foram acompanhados até a alta da unidade crítico cirúrgica. As características sociodemográficas e clínicas foram obtidas por meio de prontuários e entrevistas. Foram considerados *delirium* pós-operatório os quadros confusionais agudos identificados em até uma semana após o procedimento anestésico-cirúrgico^14^. Havia planejamento para que a obtenção de dados fosse conduzida tanto em período diurno quanto noturno, considerando que os estados confusionais agudos tendem a oscilar e a se manifestar em maior frequência durante a noite^2^. 

### Tratamento e análise dos dados

Os dados obtidos foram categorizados em fatores predisponentes (idade, sexo, histórico de cirurgia prévia, hipertensão arterial, *Diabetes Mellitus,* insuficiência renal crônica, demência, acidente vascular cerebral, acometimento psiquiátrico, alcoolismo, deficiência visual ou auditiva), e fatores precipitantes (uso de drogas ilícitas, anti-hipertensivos, antipsicóticos, antibioticoterapia, benzodiazepínicos e opioides durante o período pré-cirúrgico, caráter emergencial ou eletivo e tempo de cirurgia, uso de cristaloides, coloides, antibióticos, anticolinérgicos, analgésicos, anti-inflamatórios, antieméticos, agentes anestésicos e transfusão sanguínea).

Em seguida, as variáveis foram tabuladas pelo programa computacional *Microsoft* ^*®*^ Excel, e posteriormente analisadas pelo *software* R (versão 4.2). A associação entre os fatores selecionados e a ocorrência de *delirium* foi analisada utilizando-se o teste Exato de Fisher e o teste qui-quadrado, considerando a significância de 5%, enquanto variáveis numéricas foram comparadas por meio do Teste de Wilcoxon.

Para avaliar o efeito de cada um dos fatores analisados nas chances de ocorrência de *delirium*, foi utilizado modelo de Regressão Logística. A modelagem foi realizada em três etapas. Na primeira etapa, foi ajustado um modelo de regressão simples para cada fator analisado; na segunda etapa, foi ajustado um modelo múltiplo com as variáveis significativas a 5% na regressão simples, sendo removidas aquelas que perderam significância. Na terceira etapa, as variáveis referentes a cada um dos fatores predisponentes, precipitantes, agentes anestésicos e classes farmacológicas com significância a 20% na regressão simples foram avaliados em modelos múltiplos, sendo mantidos apenas aqueles com significância de 5%. O método de modelagem adotado por este estudo preconiza um modelo de seleção proposital onde, inicialmente, ajusta-se um modelo múltiplo incluindo as variáveis independentes que obtiveram significância até 25% na regressão simples. Em seguida, são removidas as variáveis não significativas a 5%, uma de cada vez e, após contemplar todas as variáveis, testa-se o efeito do retorno de cada variável retirada do modelo inicial com a finalidade de verificar se a variável independente removida anteriormente pode adotar um comportamento diferencial na presença de outras variáveis^15^.

### Aspectos éticos

O projeto obteve a aprovação no Comitê de Ética e Pesquisa sob parecer nº 5.051.627 e foi conduzido em caráter voluntário e confidencial. Os participantes foram abordados anteriormente ao procedimento anestésico-cirúrgico, estando em estado cognitivo preservado, e informados sobre os objetivos, a metodologia, os riscos potenciais derivados de sua participação e a possibilidade de deixar o estudo a qualquer momento.

Os riscos inerentes a esta pesquisa foram considerados baixos, pois os dados coletados integravam a rotina da unidade e a equipe de pesquisa são profissionais que atuam no setor. Para mitigar o risco relacionado ao uso indevido de informações dos participantes, foi oferecido treinamento à equipe de pesquisa.

## Resultados

Foram atendidos 236 pacientes no setor no período, dos quais 71 foram excluídos devido aos critérios de exclusão e oito, mesmo em preservado estado cognitivo, se recusaram a participar da pesquisa, totalizando 157 participantes. Conforme evidenciado na Tabela 1, os participantes possuíam mediana da idade em 63 anos, variando de 23 a 88 anos, predomínio do sexo feminino (n=84; 54%) e de hipertensos (n=104; 67%). Foram constatadas ainda as seguintes doenças crônicas: *Diabetes Mellitus* (n=57; 37%), alcoolismo (n=28; 18%), doença psiquiátrica (n=17; 11%), insuficiência renal crônica (n=11; 7,1%), acidente vascular encefálico (n=10; 6,4%) e demência (n=3; 1,9%). Houve relato quanto ao uso prévio de anti-hipertensivos (n=96; 61,1%), antipsicóticos (n=21; 13,4%), antibióticos (n=10; 6,7%), opioides (n=11; 12,6%), benzodiazepínicos (n=12; 13,8%) e drogas ilícitas (n=3; 1,9%).

**Tabela 1:** Fatores precipitantes e predisponentes para *delirium* em pacientes críticos adultos de um hospital universitário. Rio de Janeiro, RJ, Brasil, 2023

Total			*Delirium*				Regressão Simples			
Variáveis	n	n = 157*	n	Não (n = 113)*	Sim (n = 44)*	p-valor	n	OR^†^	95% IC^‡^	p-valor
**Sexo**	157		157			0.365^§^	157			
Feminino		84 (54%)		63 (75%)	21 (25%)			-	-	
Masculino		73 (46%)		50 (68%)	23 (32%)			1.38	0.69, 2.79	0.4
**Idade**	157	63 (50, 71)^||^	157	60 (48, 69)^||^	70 (60, 74)^||^	0.001^¶^	157	1.04	1.01, 1.07	0.005
**Tempo de cirurgia**	143	240 (158, 302)^||^	143	200 (148, 280)^||^	270 (218, 394)^||^	<0.001^¶^	143	1.00	1.00, 1.01	0.001
**Hipertensão arterial**	156		156			0.166^§^	156			
Não		52 (33%)		41 (79%)	11 (21%)			-	-	
Sim		104 (67%)		71 (68%)	33 (32%)			1.73	0.81, 3.92	0.2
***Diabetes Mellitus***	156		156			0.147^§^	156			
Não		99 (63%)		75 (76%)	24 (24%)			-	-	
Sim		57 (37%)		37 (65%)	20 (35%)			1.69	0.83, 3.45	0.15
**Demência**	156		156			0.192**	156			
Não		153 (98%)		111 (73%)	42 (27%)			-	-	
Sim		3 (1.9%)		1 (33%)	2 (67%)			5.29	0.49, 116	0.2
**Alcoolismo**	154		154			0.138^§^	154			
Não		126 (82%)		94 (75%)	32 (25%)			-	-	
Sim		28 (18%)		17 (61%)	11 (39%)			1.90	0.79, 4.45	0.14
**Déficit visual**	156		156			0.116^§^	156			
Não		83 (53%)		64 (77%)	19 (23%)			-	-	
Sim		73 (47%)		48 (66%)	25 (34%)			1.75	0.87, 3.58	0.12
**Déficit auditivo**	155		155			0.223**	155			
Não		141 (91%)		103 (73%)	38 (27%)			-	-	
Sim		14 (9.0%)		8 (57%)	6 (43%)			2.03	0.63, 6.23	0.2
**Transfusão intraoperatório**	153		153			0.043**	153			
Não		136 (89%)		101 (74%)	35 (26%)			-	-	
Sim		17 (11%)		8 (47%)	9 (53%)			3.25	1.16, 9.29	0.025
**Cristaloide intraoperatório**	157		157			0.008^§^	157			
Não		73 (46%)		60 (82%)	13 (18%)			-	-	
Sim		84 (54%)		53 (63%)	31 (37%)			2.70	1.30, 5.84	0.009
**Coloide intraoperatório**	157		157			0.252**	157			
Não		140 (89%)		103 (74%)	37 (26%)			-	-	
Sim		17 (11%)		10 (59%)	7 (41%)			1.95	0.66, 5.45	0.2
**Antibiótico intraoperatório**	157		157			1.000**	157			
Não		147 (94%)		106 (72%)	41 (28%)			-	-	
Sim		10 (6.4%)		7 (70%)	3 (30%)			1.11	0.23, 4.20	0.9
**Anticolinérgico intraoperatório**	153		153			0.343^§^	153			
Sim		28 (18%)		22 (79%)	6 (21%)			-	-	
Não		125 (82%)		87 (70%)	38 (30%)			1.60	0.63, 4.63	0.3
**Anti-inflamatório intraoperatório**	153		153			0.037^§^	153			
Sim		30 (20%)		26 (87%)	4 (13%)			-	-	
Não		123 (80%)		83 (67%)	40 (33%)			3.13	1.13, 11.1	0.045
**Antiemético intraoperatório**	152		152			0.129^§^	152			
Sim		97 (64%)		73 (75%)	24 (25%)			-	-	
Não		55 (36%)		35 (64%)	20 (36%)			1.74	0.85, 3.57	0.13
**Lidocaína intraoperatório**	152		152			0.139^§^	152			
Não		66 (43%)		51 (77%)	15 (23%)			-	-	
Sim		86 (57%)		57 (66%)	29 (34%)			1.73	0.84, 3.65	0.14
**Sufentanil intraoperatório**	152		152			0.117**	152			
Não		138 (91%)		101 (73%)	37 (27%)			-	-	
Sim		14 (9.2%)		7 (50%)	7 (50%)			2.73	0.88, 8.49	0.077
**Midazolam intraoperatório**	152		152			0.100^§^	152			
Sim		117 (77%)		87 (74%)	30 (26%)			-	-	
Não		35 (23%)		21 (60%)	14 (40%)			1.93	0.86, 4.26	0.10
**Propofol intraoperatório**	152		152			0.230^§^	152			
Sim		120 (79%)		88 (73%)	32 (27%)			-	-	
Não		32 (21%)		20 (62%)	12 (38%)			1.65	0.71, 3.73	0.2
**Sevoflurano intraoperatório**	153		153			0.196^§^	153			
Não		112 (73%)		83 (74%)	29 (26%)			-	-	
Sim		41 (27%)		26 (63%)	15 (37%)			1.65	0.76, 3.53	0.2

*n = Número (%); ^†^OR = *Odds Ratio*; ^‡^IC = Intervalo de Confiança; ^§^Teste qui-quadrado de Pearson; ^||^Mediana (IQR: Intervalo Interquartil); ^¶^Teste de Wilcoxon; ^**^Teste Exato de Fisher

No que tange ao procedimento anestésico-cirúrgico, a mediana do tempo intraoperatório foi 240 minutos, sendo o menor tempo 20 e o maior 705 minutos. Observou-se dominância quanto ao uso de cristaloides intravenosos (n=84; 54%), em contraste com a infusão de coloides (n=17; 11%), e houve necessidade transfusional durante o ato cirúrgico para 17 participantes (11%). Verificou-se a administração dos seguintes medicamentos durante o ato cirúrgico: analgésicos (n=119; 78%), antieméticos (n=97; 64%), anti-inflamatórios (n=30; 20%) e anticolinérgicos (n=28; 18%).

Os participantes possuíam histórico de cirurgias prévias (n=122; 77,7%) em caráter eletivo (n=145; 93,5%) e foram submetidos às seguintes especialidades: cirurgia geral (n=51; 32,6%), torácica (n=39; 25%), vascular (n=28; 17,9%), urológica (n=6; 3,8%), ginecológica (n=5; 3,2%); ortopédica (n=3; 1,9%) cabeça e pescoço (n=1; 0,6%), neurocirurgia (n=1; 0,6%). Foram empregadas diversificadas técnicas anestésicas: anestesia geral (n=88; 56%), inalatória (n=35; 22,2%), geral combinada com peridural (n=27; 17,2%), local (n=3; 1,9%), epidural (n=2; 1,2%), bloqueio subaracnoideo (n=1; 0,6%) ou exclusivamente sedação (n=1; 0,6%).

A incidência de *delirium* para a população estudada foi 28% (n=44). A idade avançada consistiu em um fator predisponente de significância estatística (p=0,001) e esta constatação se deu por meio de uma diferença expressiva entre a mediana de idade para participantes delirantes (70 anos) comparada aos não delirantes (60 anos). No que se refere aos fatores precipitantes avaliados durante o período intraoperatório, evidenciou-se significância quanto ao tempo de cirurgia (p<0,001), o emprego de transfusão sanguínea (p=0,043), a administração de cristaloides (p=0,008) e anti-inflamatórios (p=0,037) para o desfecho *delirium*.

Ajustou-se um modelo múltiplo com as variáveis significativas a 5%, sendo removidas aquelas que perderam significância, resultando um modelo com a idade e o tempo de cirurgia. A partir deste, foram adicionadas as variáveis significativas a 20%, e obteve-se um modelo ajustado composto pelas variáveis: idade, tempo de cirurgia, administração de antieméticos, o uso de sufentanil e transfusão sanguínea durante o período intraoperatório, conforme apresentado na Tabela 2. 

**Tabela 2:** Modelo Multivariado de Regressão Logística dos fatores precipitantes e predisponentes para o *delirium* em pacientes críticos adultos de um hospital universitário. Rio de Janeiro, RJ, Brasil, 2023

	Modelo Inicial			Modelo Final		
Característica	OR*	95% IC^†^	p-valor	OR*	95% IC^†^	p-valor
**Idade**	1.038	1.011, 1.068	0.008	1.037	1.010, 1.069	0.010
**Tempo de cirurgia**	1.005	1.002, 1.008	0.001	1.004	1.001, 1.008	0.005
**Antiemético intraoperatório**						
Sim				-	-	
Não				2.895	1.196, 7.229	0.020
**Sufentanil intraoperatório**						
Não				-	-	
Sim				5.748	1.488, 23.66	0.012
**Transfusão intraoperatório**						
Não				-	-	
Sim				3.814	1.064, 14.29	0.041

*OR = *Odds Ratio*; ^†^IC = Intervalo de Confiança

## Discussão

A incidência de *delirium* pós-operatório (28%) está moderadamente acima da literatura consultada, que aponta 24% para idosos submetidos às cirurgias não cardíacas^16^ e 26,6% para idosos submetidos aos procedimentos ortopédicos^8^. 

Verifica-se neste estudo que o incremento na idade (OR: 1,037). Apesar de a literatura apontar o impacto do histórico de saúde para o desenvolvimento de *delirium* ^10^, esta associação não foi evidenciada neste estudo. No entanto, constatou-se que a idade representa um fator predisponente significativo e que cada ano de vida aumenta o risco para *delirium* em 3,7%. Isto se justifica devido à apoptose de neurônios, à diminuição do fluxo sanguíneo cerebral e às alterações no sistema neurotransmissor^4^.

A duração do procedimento anestésico-cirúrgico se mostrou significativo e cada minuto cirúrgico aumenta a chance para *delirium* em 0,4%. Este dado está em consonância com outros autores, que destacam a maior incidência de *delirium* em pacientes submetidos às cirurgias torácicas cuja duração seja superior a 80 minutos^17^, justificando ainda a preconização para que as cirurgias de cabeça e pescoço sejam realizadas por dois cirurgiões, tendo por objetivo abreviar a duração do procedimento^18^.

Os anti-inflamatórios identificados durante o ato cirúrgico consistem em medicamentos inibidores seletivos da cicloxigenase-2 (parecoxibe) e não seletivos (cetoprofeno), e foram associados significativamente ao DPO. Embora o impacto quanto ao uso desta classe farmacológica não tenha sido elucidado, suscita-se que as citocinas, compreendidas como um agregado de mediadores celulares peptídeos produzidos a partir de uma resposta inflamatória, possam ter papel desencadeador para o *delirium* decorrente do aumento da permeabilidade da barreira hematoencefálica^19^. 

A variável de maior impacto foi o uso intraoperatório de sufentanil, opioide com potência cinco a 10 vezes maior quando comparado ao fentanil^20^, resultando em um aumento de 474% em chance para a ocorrência de *delirium* (OR: 5,748). Este achado é corroborado por outro estudo de coorte prospectivo em pacientes submetidos à prostatectomia radical eletiva^9^. Um desafio quanto ao emprego de opiáceos e desenvolvimento de *delirium* consiste na determinação da dose eficaz. A literatura destaca que a dor representa um fator de risco elevado e incrementa a chance para ocorrência de *delirium* (9,85 vezes), o que aponta para a necessidade de uma condução sistematizada para a avaliação e o manejo da dor perioperatória^21^. 

Este estudo revela ainda que 53% dos que receberam transfusão sanguínea durante o intraoperatório apresentaram *delirium*, o que reflete em aumento de 281% na chance de ocorrência do *delirium* (OR: 3,814). A anemia consiste em um dos fatores precipitantes mais preditivos^21^ e o sangramento intraoperatório é considerado fator de risco para *delirium*, sobretudo quando ultrapassa a perda de 400 ml^8^.

Durante o intraoperatório, a reposição volêmica tem por objetivo promover a manutenção hídrica ou hemodinâmica^22^. No entanto, usualmente realizada por meio de administração de cristaloides, seja por emprego de solução fisiológica ou solução de *Ringer*, esta terapêutica propicia a queda da pressão coloidosmótica plasmática, resulta na perda de fluidos para o terceiro espaço. Nesse contexto, um estudo sobre a prevenção de hipotensão durante raquianestesia durante cesariana evidenciou que menos pacientes apresentaram hipotensão no grupo coloide em comparação ao grupo cristaloide^22^. 

Observa-se que a literatura aponta causas secundárias para a necessidade de reposição volêmica como fatores precipitantes para o *delirium*, como a associação entre *delirium* e hipotensão durante o procedimento anestésico-cirúrgico^23^ e desidratação durante o período pós-operatório^21^. No entanto, este estudo se diferencia por avaliar a natureza da reposição hídrica, ressaltando o emprego de cristaloides como evento significativo quando comparado ao uso de coloides. 

Ainda que o manejo farmacológico para o *delirium* não tenha se mostrado como uma estratégia eficaz, alguns estudos sobre a prevenção ou tratamento do *delirium* pós-cirúrgico têm focado na possibilidade da ondansetrona ser um fármaco promissor. O *delirium* é uma manifestação importante da síndrome serotoninérgica e a ondansetrona consiste em um antagonista da serotonina específico para o receptor 5-HT3 e, rotineiramente, é empregada durante o período pós-operatório para o controle de náuseas e vômitos^24^.

Embora este estudo não tenha o intuito de apresentar a ondansetrona como estratégia farmacológica para o manejo do *delirium*, destaca-se que a não utilização deste fármaco durante o período intraoperatório mostrou-se associado a um incremento de 198% (OR: 2,895) na chance de ocorrência de *delirium*. Portanto, este achado possui relevância por fornecer subsídios fomentadores da hipótese relacional dos neurotransmissores deflagrantes de *delirium*, sobretudo a serotonina.

Entretanto, ainda que esta pesquisa tenha evidenciado a associação de fatores predisponentes e precipitantes com o *delirium*, algumas limitações devem ser explicitadas, como a não individualização referente às doses dos medicamentos avaliados, bem como o cenário de estudo tratar-se de um hospital universitário de alta complexidade e que não possui atendimento emergencial, suscitando a possibilidade de que a população estudada possua características cirúrgicas diferenciadas, e que os achados possam não ser generalizáveis. 

Além disso, destaca-se a impossibilidade para a obtenção de algumas variáveis de estudo para todos os participantes, que foram tabuladas como dados faltantes devido à incompletude de informações em prontuários. Outras não puderam ser avaliadas por este estudo, como o estado nutricional prévio e a classificação de risco cirúrgico conforme *American Society of Anesthesiology* (ASA), dados considerados relevantes para a ocorrência de *delirium*, porém ausentes nos prontuários dos participantes.

Ressalta-se que, embora exista uma correlação entre os fatores precipitantes e predisponentes com o *delirium* pós-operatório, entende-se que esta não consolide casualidade e que os fatores associados possam estar relacionados a uma causa subjacente ainda não elucidada na literatura.

Nesse caso, suscita-se que a identificação dos fatores de risco realizado por este estudo contribua para o direcionamento científico sobre a elucidação fisiopatológica do *delirium* e substancie, também, publicações futuras voltadas à validação de intervenções que visem reduzir o risco para DPO.

## Conclusão

Constatou-se que o *delirium* representa um acometimento frequente durante o período pós-operatório de adultos críticos. Tendo em vista a terapia intensiva como um ambiente propício para tal, seja devido à situação de confinamento subjacente ou pela condição orgânica desfavorável de seus pacientes, e tendo como evento catalisador o procedimento anestésico-cirúrgico, postula-se como estratégia mitigadora a identificação de fatores associados ao *delirium*.

Ainda que alguns fatores predisponentes para o *delirium* sejam bem determinados na literatura, este estudo se diferencia por estabelecer os fatores precipitantes relacionados ao período intraoperatório, como o tempo cirúrgico, o uso de sufentanil, o emprego de transfusão sanguínea, a infusão de agentes anestésicos, bem como a não administração de ondansetrona, um antagonista da serotonina e corrobora, portanto, a hipótese relacional de neurotransmissores para o desenvolvimento de *delirium*.

Desse modo, destaca-se a atuação do enfermeiro nas terapias intensivas cirúrgicas, tanto para a identificação do *delirium* por meio do emprego de instrumentos validados quanto para a detecção de fatores associados, sobretudo, em idosos e pacientes que se submeteram às cirurgias prolongadas, os quais têm maiores chances de desenvolver esta disfunção. Com isso, pretende-se oferecer subsídios que norteiam a sistematização da assistência de Enfermagem perioperatória, na medida em que este trabalho agrega complementaridade ao diagnóstico de Enfermagem denominado confusão aguda.

Postula-se que este estudo propicie uma vasta contribuição social na medida em que aborda uma entidade nosológica frequente em idosos em um panorama global de envelhecimento populacional. Acredita-se, ainda, na redução dos custos socioeconômicos em consequência do aprimoramento da qualidade dos cuidados de saúde, bem como o aumento da expectativa de vida e fomentação para a implementação de tecnologias de cuidado inovadoras.

# Translation: Factores predisponentes y precipitantes para el desarrollo del *delirium* postoperatorio en pacientes críticos de una unidad intensiva universitaria

**DOI:** 10.1590/1518-8345.7113.4232

**Authors:** Danielle Moreira Marques, Davi da Silveira Barroso Alves, Taís Veronica Cardoso Vernaglia

**Affiliations:** 1 Universidade Federal do Estado do Rio de Janeiro, Rio de Janeiro, RJ, Brasil.; 2 Universidade do Estado do Rio de Janeiro, Hospital Universitário Pedro Ernesto, Rio de Janeiro, RJ, Brasil.; 3 Universidade Federal do Estado do Rio de Janeiro, Departamento de Métodos Quantitativos, Rio de Janeiro, RJ, Brasil.; 4 Universidade Federal do Estado do Rio de Janeiro, Escola de Enfermagem Alfredo Pinto, Rio de Janeiro, RJ, Brasil.

**Keywords:** Delirium, Delirio del Despertar, Enfermería en Neurociencias, Cuidados Posoperatorios, Medidas de Asociación, Exposición, Riesgo o Desenlace, Cuidados Críticos

## Abstract

**Objetivo:**

detectar la incidencia de *delirium* postoperatorio en pacientes críticos internados en una unidad de terapia intensiva quirúrgica y evaluar los factores predisponentes y precipitantes asociados al *delirium* postoperatorio en pacientes críticos internados en una unidad de terapia intensiva quirúrgica.

**Método:**

se trata de un estudio de cohorte prospectivo con 157 pacientes críticos quirúrgicos. Se utilizó la prueba exacta de Fisher y chi-cuadrado para la asociación entre los factores y ocurrencia de *delirium*, prueba de Wilcoxon para las variables numéricas y el modelo de regresión logística para el análisis de los factores predisponentes y precipitantes.

**Resultados:**

la incidencia de *delirium* fue 28% (n=44). El factor edad se presentó como predisponente significativo (p=0,001), seguido del tiempo de cirugía (p<0,001), la transfusión sanguínea (p=0,043), la administración de cristaloides (p=0,008) y antiinflamatorios (p=0,037), estos fueron los factores precipitantes identificados. Se obtuvo como mejor modelo ajustado: edad, tiempo de cirugía, la no administración de antieméticos, el uso de sufentanilo y la transfusión sanguínea.

**Conclusión:**

el *delirium* es un acometimiento frecuente para adultos críticos quirúrgicos y la existencia de factores precipitantes y predisponentes se vuelven relevantes para el desenlace, teniendo el procedimiento anestésico-quirúrgico como evento catalizador.

## Destacados

**(1)** Elucida factores precipitantes considerados insólitos en la literatura.

**(2)** Corrobora la hipótesis relacional de neurotransmisores para la ocurrencia de *delirium*.

**(3)** Desarrolla un modelo ajustado para la identificación precoz de individuos susceptibles.

**(4)** Evidencia que el uso de opioide (sufentanilo) acarrea mayor impacto para el *delirium*.

**(5)** Agrega complementariedad al diagnóstico de Enfermería denominado confusión aguda.

## Introducción

El *delirium* consiste en una disfunción aguda y fluctuante del estado mental, caracterizada por un trastorno de la atención, conciencia y cognición, que involucra una amplia variedad de manifestaciones clínicas, con evolución versátil en el compromiso de la funcionalidad cognitiva^1^. 

Descrito como un acometimiento frecuente en pacientes críticos, su incidencia puede llegar al 80%, dependiendo de la población estudiada y los criterios diagnósticos utilizados^2^. En pacientes sometidos a procedimientos anestésico-quirúrgicos, esta condición tiende a ser subnotificada, alcanzando del 33,1 al 46,2% para aquellos sometidos a cirugías electivas o de emergencia, respectivamente^3^, y por cada día en *delirium*, hay un incremento del 10% en el riesgo de muerte^2^. 

Tal condición implica un gasto hospitalario, exclusivamente en Estados Unidos, que varía entre 806 y 24.509 dólares y, en la terapia intensiva, entre 1.529 y 14.462 por individuo^4^. Además de aumentar el tiempo de permanencia hospitalaria y los días en ventilación mecánica, el *delirium* representa un evento indeseable para los pacientes y familiares, ya que el declive cognitivo puede persistir por meses o años, acarreando un impacto en la calidad de vida, así como el compromiso social y económico^5^. 

Aunque los factores predisponentes y precipitantes no estén dilucidados por completo^5^, se plantea que el establecimiento del cuadro depende de una compleja interacción entre tales factores. Los factores predisponentes reflejan las condiciones no modificables como la edad y las comorbilidades. Ya los factores precipitantes suelen ser susceptibles de modificación y representan insultos ocasionados por la hospitalización como el empleo excesivo de tecnología, tratamiento farmacológico y alteración del ciclo sueño-vigilia^6^.

Al considerar que las estrategias farmacológicas no consisten en la primera opción terapéutica para el *delirium* y ni siempre se muestran eficaces^2^, se vuelve imprescindible identificar los factores predisponentes y precipitantes. Además, se entiende que el *delirium* consiste en una entidad nosológica representada por el diagnóstico de Enfermería denominado *confusión aguda*, definido por perturbaciones reversibles de conciencia, atención, cognición y percepción que se desarrollan en un corto período^7^. 

Aunque los cuadros confusionales agudos derivan de alteraciones orgánicas, estos se manifiestan, sobre todo, por alteraciones conductuales reconocidas por el enfermero. Este profesional actúa en la planificación y en la implementación de intervenciones dirigidas a los factores de riesgo identificados, así como en la prevención y en la detección precoz del *delirium*, evaluación de los resultados y evolución de los cuidados determinados^7^. 

Algunos estudios revelan que hay mayor probabilidad para el desarrollo de *delirium* durante el período postoperatorio de individuos más viejos^4^. Sin embargo, no hay consenso en relación al impacto del evento quirúrgico para el desarrollo del *delirium*. Por ejemplo, se destaca la anestesia general, en la cual el empleo aislado denota mayor incidencia de declive cognitivo cuando se compara a los pacientes que la utilizan en carácter regional^8^ y el prolongamiento del procedimiento anestésico-quirúrgico^9^, los cuales fueron evidenciados en la literatura como factores correlacionados al *delirium*. En contraste, otros autores consideraron que los factores predisponentes relacionados a las enfermedades de base, como la demencia y la insuficiencia cardíaca, denotan factores de mayor impacto^10^.

De este modo, al comprender la brecha científica sobre la elucidación del *delirium* asociado al evento anestésico-quirúrgico y el papel del enfermero para la identificación de factores desencadenantes y planificación de acciones mitigadoras, se tienen por objetivos detectar la incidencia de *delirium* postoperatorio en pacientes críticos internados en una unidad de terapia intensiva quirúrgica; y evaluar los factores predisponentes y precipitantes asociados al *delirium* postoperatorio en pacientes críticos internados en una unidad de terapia intensiva quirúrgica.

## Método

### Tipo de estudio

Se trata de un estudio de cohorte prospectivo y de enfoque cuantitativo.

### Escenario

La investigación se llevó a cabo en una unidad crítico quirúrgica de un hospital universitario ubicado en el municipio de Río de Janeiro, en el estado de Río de Janeiro, Brasil. La unidad está compuesta por siete camas de internación destinadas a individuos masculinos y femeninos, sometidos a procedimientos quirúrgicos no cardiológicos de gran envergadura. Este hospital realiza, en promedio, 35 internaciones mensuales, con un tiempo medio de permanencia que consiste en 2,3 días. El sector asegura la visita familiar diaria en horarios predeterminados, y posee infraestructura adecuada para la oferta de asistencia de calidad y seguridad a las personas en estado crítico.

### Período

El estudio se realizó en el período del cinco de enero al cuatro de julio de 2022.

### Población

La población del estudio estuvo compuesta por 157 pacientes elegibles que aceptaron participar en la investigación, y que fueron seguidos integralmente durante el período de recolección de datos. Para este estudio, no hubo pérdida de seguimiento.

### Criterios de selección

Se incluyeron los pacientes durante el período postoperatorio inmediato en unidad crítico quirúrgica no cardiológica, mayores de 18 años y se consideraron como criterio de exclusión los pacientes sometidos a cirugía intracraneal, comprendiendo que esta modalidad quirúrgica puede representar un sesgo confusional para la evaluación neurológica.

### Participantes

Considerando el promedio de 35 internaciones mensuales en el escenario de estudio y el tiempo de seguimiento determinado, se obtuvo una estimación de 210 internaciones en seis meses. Para la definición del tamaño de la muestra, se adoptó el Intervalo de Confianza (IC) del 95% y un margen de error máximo para la proporción del 5%, obteniéndose 137 participantes^11^. Cabe destacar que no se realizó el proceso de muestreo para la selección de los participantes, utilizando el cálculo muestral como subsidio para fundamentar el período de seguimiento del estudio.

### Instrumentos utilizados para la recolección de las informaciones

Se realizó una revisión de la literatura sobre los factores de riesgo para el desarrollo de *delirium* postoperatorio (DPO) con fines de definición de las variables que compusieron el instrumento de recolección de datos, basado en los siguientes ejes: datos sociodemográficos, historial de salud, ocurrencias intraoperatorias y seguimiento postquirúrgico en la terapia intensiva.

La ocurrencia del *delirium* fue constatada por el empleo del *Confusion Assessment Method for the Intensive Care Unit* (CAM-ICU) - método validado para la identificación de *delirium*. Esta herramienta, que fue elaborada en 2001, requiere la utilización de la Escala de Agitación y Sedación de Richmond (RASS), y está compuesta por la evaluación de los ítems: inicio agudo o curso fluctuante del cuadro, trastorno de la atención, pensamiento desorganizado y alteración del nivel de consciencia^12^. El instrumento está validado para el idioma portugués y posee alta sensibilidad y especificidad. Su empleo se da al lado de la cama y exige entrenamiento mínimo^13^.

### Recolección de datos

La recolección de datos fue realizada por trece enfermeros que trabajaban en el escenario de estudio, durante el período de recolección de datos. Son profesionales con más de dos años de experiencia en terapias intensivas, y que recibieron capacitación por parte de la investigadora principal responsable del estudio para la evaluación y aplicación de los instrumentos de recolección de datos. La capacitación consistió en la presentación de los instrumentos para la recolección de información, aclaración sobre los aspectos éticos y las etapas para la aplicación del método CAM-ICU. 

Los participantes fueron abordados inmediatamente a su admisión y fueron seguidos hasta el alta de la unidad crítico quirúrgica. Las características sociodemográficas y clínicas se obtuvieron mediante historiales médicos y entrevistas. Se consideraron *delirium* pós-operatório los cuadros confusionales agudos identificados hasta una semana después del procedimiento anestésico-quirúrgico^14^. Se planificó que la obtención de datos se llevara a cabo tanto en el período diurno como en el nocturno, considerando que los estados confusionales agudos tienden a fluctuar y manifestarse con mayor frecuencia durante la noche^2^.

### Tratamiento y análisis de los datos

Los datos obtenidos fueron categorizados en factores predisponentes (edad, sexo, historial de cirugía previa, hipertensión arterial, *Diabetes Mellitus*, insuficiencia renal crónica, demencia, accidente cerebrovascular, afección psiquiátrica, alcoholismo, deficiencia visual o auditiva) y factores precipitantes (uso de drogas ilícitas, antihipertensivos, antipsicóticos, antibioticoterapia, benzodiacepinas y opioides durante el período prequirúrgico, carácter emergencial o electivo y tiempo de cirugía, uso de cristaloides, coloides, antibióticos, anticolinérgicos, analgésicos, antiinflamatorios, antieméticos, agentes anestésicos y transfusión sanguínea). 

A continuación, las variables se tabularon utilizando el programa computacional *Microsoft* ^*®*^ Excel, y posteriormente se analizaron con el *software* R (versión 4.2). La asociación entre los factores seleccionados y la ocurrencia de *delirium* se analizó utilizando la prueba exacta de Fisher y la prueba de chi-cuadrado, considerando una significancia del 5%, mientras que las variables numéricas se compararon mediante la prueba de *Wilcoxon*. 

Para evaluar el efecto de cada uno de los factores analizados en las probabilidades de ocurrencia de *delirium*, se utilizó un modelo de regresión logística. La modelización se realizó en tres etapas. En la primera etapa, se ajustó un modelo de regresión simple para cada factor analizado; en la segunda etapa, se ajustó un modelo múltiple con las variables significativas al 5% en la regresión simple, siendo eliminadas aquellas que perdieron significancia. En la tercera etapa, las variables referentes a cada uno de los factores predisponentes, precipitantes, agentes anestésicos y clases farmacológicas con significancia al 20% en la regresión simple fueron evaluadas en modelos múltiples, manteniéndose solo aquellas con una significancia del 5%. El método de modelado adoptado por este estudio preconiza un modelo de selección intencionada donde, inicialmente, se ajusta un modelo múltiple incluyendo las variables independientes que obtuvieron significancia hasta el 25% en la regresión simple. A continuación, se eliminan las variables no significativas al 5%, una a una y, después de considerar todas las variables, se prueba el efecto del retorno de cada variable retirada del modelo inicial con el fin de verificar si la variable independiente eliminada anteriormente puede adoptar un comportamiento diferencial en presencia de otras variables.

### Aspectos éticos

El proyecto obtuvo la aprobación del Comité de Ética e Investigación bajo el dictamen nº 5.051.627 y se llevó a cabo de manera voluntaria y confidencial. Los participantes fueron abordados previamente al procedimiento anestésico-quirúrgico, estando en estado cognitivo preservado, e informados sobre los objetivos, la metodología, los riesgos potenciales derivados de su participación y la posibilidad de abandonar el estudio en cualquier momento.

Los riesgos inherentes a esta investigación se consideraron bajos, ya que los datos recopilados formaban parte de la rutina de la unidad y el equipo de investigación son profesionales que trabajan en el sector. Para mitigar el riesgo relacionado con el uso indebido de información de los participantes, se ofreció capacitación al equipo de investigación.

## Resultados

Se atendieron 236 pacientes en el sector en el período, de los cuales 71 fueron excluidos debido a los criterios de exclusión y ocho, aun estando en estado cognitivo preservado, se negaron a participar en la investigación, sumando un total de 157 participantes. Conforme se evidencia en la Tabla 1, los participantes tenían una mediana de edad de 63 años, variando de 23 a 88 años, predominio del sexo femenino (n=84; 54%) y de hipertensos (n=104; 67%). También se constataron las siguientes enfermedades crónicas: *Diabetes Mellitus* (n=57; 37%), alcoholismo (n=28; 18%), enfermedad psiquiátrica (n=17; 11%), insuficiencia renal crónica (n=11; 7,1%), accidente cerebrovascular (n=10; 6,4%) y demencia (n=3; 1,9%). Hubo reportes sobre el uso previo de antihipertensivos (n=96; 61,1%), antipsicóticos (n=21; 13,4%), antibióticos (n=10; 6,7%), opioides (n=11; 12,6%), benzodiacepinas (n=12; 13,8%) y drogas ilícitas (n=3; 1,9%). 

**Tabla 1:** Factores precipitantes y predisponentes al *delirium* en pacientes adultos críticos en un hospital universitario. Río de Janeiro, RJ, Brasil, 2023

Total			*Delirium*				Regresión simple			
Variables	n	n = 157*	n	No (n = 113)*	Sí (n = 44)*	p-valor	n	OR^†^	95% IC^‡^	p-valor
**Sexo**	157		157			0.365^§^	157			
Femenino		84 (54%)		63 (75%)	21 (25%)			-	-	
Masculino		73 (46%)		50 (68%)	23 (32%)			1.38	0.69, 2.79	0.4
**Edad**	157	63 (50, 71)^||^	157	60 (48, 69)^||^	70 (60, 74)^||^	0.001^¶^	157	1.04	1.01, 1.07	0.005
**Tiempo de cirugía**	143	240 (158, 302)^||^	143	200 (148, 280)^||^	270 (218, 394)^||^	<0.001^¶^	143	1.00	1.00, 1.01	0.001
**Hipertensión arterial**	156		156			0.166^§^	156			
No		52 (33%)		41 (79%)	11 (21%)			-	-	
Sí		104 (67%)		71 (68%)	33 (32%)			1.73	0.81, 3.92	0.2
***Diabetes Mellitus***	156		156			0.147^§^	156			
No		99 (63%)		75 (76%)	24 (24%)			-	-	
Sí		57 (37%)		37 (65%)	20 (35%)			1.69	0.83, 3.45	0.15
**Demencia**	156		156			0.192**	156			
No		153 (98%)		111 (73%)	42 (27%)			-	-	
Sí		3 (1.9%)		1 (33%)	2 (67%)			5.29	0.49, 116	0.2
**Alcoholismo**	154		154			0.138^§^	154			
No		126 (82%)		94 (75%)	32 (25%)			-	-	
Sí		28 (18%)		17 (61%)	11 (39%)			1.90	0.79, 4.45	0.14
**Déficit visual**	156		156			0.116^§^	156			
No		83 (53%)		64 (77%)	19 (23%)			-	-	
Sí		73 (47%)		48 (66%)	25 (34%)			1.75	0.87, 3.58	0.12
**Déficit de audición**	155		155			0.223**	155			
No		141 (91%)		103 (73%)	38 (27%)			-	-	
Sí		14 (9.0%)		8 (57%)	6 (43%)			2.03	0.63, 6.23	0.2
**Transfusión intraoperatoria**	153		153			0.043**	153			
No		136 (89%)		101 (74%)	35 (26%)			-	-	
Sí		17 (11%)		8 (47%)	9 (53%)			3.25	1.16, 9.29	0.025
**Cristaloide intraoperatorio**	157		157			0.008^§^	157			
No		73 (46%)		60 (82%)	13 (18%)			-	-	
Sí		84 (54%)		53 (63%)	31 (37%)			2.70	1.30, 5.84	0.009
**Coloide intraoperatorio**	157		157			0.252**	157			
No		140 (89%)		103 (74%)	37 (26%)			-	-	
Sí		17 (11%)		10 (59%)	7 (41%)			1.95	0.66, 5.45	0.2
**Antibiótico intraoperatorio**	157		157			1.000**	157			
No		147 (94%)		106 (72%)	41 (28%)			-	-	
Sí		10 (6.4%)		7 (70%)	3 (30%)			1.11	0.23, 4.20	0.9
**Anticolinérgico intraoperatorio**	153		153			0.343^§^	153			
Sí		28 (18%)		22 (79%)	6 (21%)			-	-	
No		125 (82%)		87 (70%)	38 (30%)			1.60	0.63, 4.63	0.3
**Antiinflamatorio intraoperatorio**	153		153			0.037^§^	153			
Sí		30 (20%)		26 (87%)	4 (13%)			-	-	
No		123 (80%)		83 (67%)	40 (33%)			3.13	1.13, 11.1	0.045
**Antiemético intraoperatorio**	152		152			0.129^§^	152			
Sí		97 (64%)		73 (75%)	24 (25%)			-	-	
No		55 (36%)		35 (64%)	20 (36%)			1.74	0.85, 3.57	0.13
**Lidocaína intraoperatório**	152		152			0.139^§^	152			
No		66 (43%)		51 (77%)	15 (23%)			-	-	
Sí		86 (57%)		57 (66%)	29 (34%)			1.73	0.84, 3.65	0.14
**Sufentanilo intraoperatorio**	152		152			0.117**	152			
No		138 (91%)		101 (73%)	37 (27%)			-	-	
Sí		14 (9.2%)		7 (50%)	7 (50%)			2.73	0.88, 8.49	0.077
**Midazolam intraoperatorio**	152		152			0.100^§^	152			
Sí		117 (77%)		87 (74%)	30 (26%)			-	-	
No		35 (23%)		21 (60%)	14 (40%)			1.93	0.86, 4.26	0.10
**Propofol intraoperatorio**	152		152			0.230^§^	152			
Sí		120 (79%)		88 (73%)	32 (27%)			-	-	
No		32 (21%)		20 (62%)	12 (38%)			1.65	0.71, 3.73	0.2
**Sevoflurano intraoperatorio**	153		153			0.196^§^	153			
No		112 (73%)		83 (74%)	29 (26%)			-	-	
Sí		41 (27%)		26 (63%)	15 (37%)			1.65	0.76, 3.53	0.2

*n = Número (%); ^†^OR = *Odds Ratio*; ^‡^IC = Intervalo de Confianza; ^§^Prueba chi-cuadrado de Pearson; ^||^Mediana (IQR: Intervalo Intercuartil); ^¶^Prueba de Wilcoxon; ^**^Prueba exacta de Fisher

En cuanto al procedimiento anestésico-quirúrgico, la mediana del tiempo intraoperatorio fue de 240 minutos, siendo el menor tiempo 20 y el mayor 705 minutos. Se observó dominancia en el uso de cristaloides intravenosos (n=84; 54%), en contraste con la infusión de coloides (n=17; 11%), y hubo necesidad transfusional durante el acto quirúrgico para 17 participantes (11%). Se verificó la administración de los siguientes medicamentos durante el acto quirúrgico: analgésicos (n=119; 78%), antieméticos (n=97; 64%), antiinflamatorios (n=30; 20%) y anticolinérgicos (n=28; 18%). 

Los participantes tenían historial de cirugías previas (n=122; 77,7%) de carácter electivo (n=145; 93,5%) y fueron sometidos a las siguientes especialidades: cirugía general (n=51; 32,6%), torácica (n=39; 25%), vascular (n=28; 17,9%), urológica (n=6; 3,8%), ginecológica (n=5; 3,2%); ortopédica (n=3; 1,9%), cabeza y cuello (n=1; 0,6%), neurocirugía (n=1; 0,6%). Se emplearon diversas técnicas anestésicas: anestesia general (n=88; 56%), inhalatoria (n=35; 22,2%), general combinada con epidural (n=27; 17,2%), local (n=3; 1,9%), epidural (n=2; 1,2%), bloqueo subaracnoideo (n=1; 0,6%) o exclusivamente sedación (n=1; 0,6%). 

La incidencia de *delirium* para la población estudiada fue del 28% (n=44). La edad avanzada consistió en un factor predisponente de significancia estadística (p=0,001) y esta constatación se dio por medio de una diferencia significativa entre la mediana de edad para participantes delirantes (70 años) comparada con los no delirantes (60 años). En lo que respecta a los factores precipitantes evaluados durante el período intraoperatorio, se evidenció significancia en cuanto al tiempo de cirugía (p<0,001), el empleo de transfusión sanguínea (p=0,043), la administración de cristaloides (p=0,008) y antiinflamatorios (p=0,037) para el desenlace *delirium*.

Se ajustó un modelo múltiple con las variables significativas al 5%, siendo eliminadas aquellas que perdieron significancia, resultando en un modelo con la edad y el tiempo de cirugía. A partir de este, se añadieron las variables significativas al 20%, y se obtuvo un modelo ajustado compuesto por las variables: edad, tiempo de cirugía, administración de antieméticos, el uso de sufentanilo y transfusión sanguínea durante el período intraoperatorio, como se presenta en la Tabla 2.

**Tabla 2:** Modelo de regresión logística multivariado de factores precipitantes y predisponentes al *delirium* en pacientes críticos adultos de un hospital universitario. Río de Janeiro, RJ, Brasil, 2023

	Modelo Inicial			Modelo Final		
Característica	OR*	95% IC^†^	p-valor	OR*	95% IC^†^	p-valor
**Edad**	1.038	1.011, 1.068	0.008	1.037	1.010, 1.069	0.010
**Tiempo de cirurgía**	1.005	1.002, 1.008	0.001	1.004	1.001, 1.008	0.005
**Antiemético intraoperatorio**						
Sí				-	-	
No				2.895	1.196, 7.229	0.020
**Sufentanilo intraoperatorio**						
No				-	-	
Sí				5.748	1.488, 23.66	0.012
**Transfusión intraoperatoria**						
No				-	-	
Sí				3.814	1.064, 14.29	0.041

*OR = *Odds Ratio*; ^†^IC = Intervalo de Confianza

## Discusión

La incidencia de *delirium* postoperatorio (28%) está moderadamente por encima de la literatura consultada, que indica un 24% para personas mayores sometidas a cirugías no cardíacas^16^ y un 26,6% para adultos mayores sometidos a procedimientos ortopédicos^8^. 

Se verifica en este estudio que el incremento en la edad (OR: 1,037). A pesar de que la literatura señala el impacto del historial de salud para el desarrollo de *delirium* ^10^, esta asociación no fue evidenciada en este estudio. No obstante, se constató que la edad representa un factor predisponente significativo y que cada año de vida aumenta el riesgo para *delirium* en un 3,7%. Esto se justifica debido a la apoptosis de neuronas, la disminución del flujo sanguíneo cerebral y las alteraciones en el sistema neurotransmisor^4^. 

La duración del procedimiento anestésico-quirúrgico se mostró significativa y cada minuto quirúrgico aumenta la posibilidad de *delirium* en un 0,4%. Este dato está en consonancia con otros autores, que destacan la mayor incidencia de *delirium* en pacientes sometidos a cirugías torácicas cuya duración sea superior a 80 minutos^17^, justificando además, la recomendación de que las cirugías de cabeza y cuello sean realizadas por dos cirujanos, con el objetivo de abreviar la duración del procedimiento^18^. 

Los antiinflamatorios identificados durante el acto quirúrgico consisten en medicamentos inhibidores selectivos de la ciclooxigenasa-2 (parecoxib) y no selectivos (cetoprofeno), y fueron asociados significativamente al DPO. Aunque el impacto del uso de esta clase farmacológica no ha sido elucidado, se sugiere que las citocinas, comprendidas como un agregado de mediadores celulares péptidos producidos a partir de una respuesta inflamatoria, puedan tener un papel desencadenante para el *delirium* debido al aumento de la permeabilidad de la barrera hematoencefálica^19^. 

La variable de mayor impacto fue el uso intraoperatorio de sufentanilo, opioide con potencia de cinco a 10 veces mayor en comparación con el fentanilo^20^, resultando en un aumento del 474% en la posibilidad de ocurrencia de *delirium* (OR: 5,748). Este hallazgo es corroborado por otro estudio de cohorte prospectivo en pacientes sometidos a prostatectomía radical electiva^9^. Un desafío respecto al uso de opioides y el desarrollo de *delirium* consiste en la determinación de la dosis efectiva. La literatura destaca que el dolor representa un factor de riesgo elevado e incrementa la posibilidad de ocurrencia de *delirium* (9,85 veces), lo que señala la necesidad de un manejo sistemático para la evaluación y el manejo del dolor perioperatorio^21^. 

Este estudio revela además que el 53% de los que recibieron transfusión sanguínea durante el intraoperatorio presentaron *delirium* y refleja un aumento del 281% en la posibilidad de ocurrencia de *delirium* (OR: 3,814). La anemia es uno de los factores precipitantes más predictivos^21^ y el sangrado intraoperatorio es considerado un factor de riesgo para *delirium*, especialmente, cuando supera la pérdida de 400 ml^8^.

Durante el intraoperatorio, la reposición de volumen tiene como objetivo promover el mantenimiento hídrico o hemodinámico^22^. Sin embargo, usualmente realizada mediante la administración de cristaloides, ya sea mediante el uso de solución fisiológica o solución de Ringer, esta terapéutica propicia la caída de la presión coloidosmótica plasmática, resultando en la pérdida de fluidos hacia el tercer espacio. En este contexto, un estudio sobre la prevención de hipotensión durante la raquianestesia en cesáreas, evidenció que menos pacientes presentaron hipotensión en el grupo de coloides en comparación con el grupo de cristaloides^22^. 

Se observa que la literatura señala causas secundarias para la necesidad de reposición de volumen como factores precipitantes para el *delirium*, como la asociación entre *delirium* e hipotensión durante el procedimiento anestésico-quirúrgico^23^ y deshidratación durante el período postoperatorio^21^. Sin embargo, este estudio se diferencia por evaluar la naturaleza de la reposición hídrica, resaltando el uso de cristaloides como un evento significativo en comparación con el uso de coloides. 

Aunque el manejo farmacológico para el *delirium* no se ha mostrado como una estrategia eficaz, algunos estudios sobre la prevención o tratamiento del *delirium* postquirúrgico se han centrado en la posibilidad de que el ondansetrón sea un fármaco prometedor. El *delirium* es una manifestación importante del síndrome serotoninérgico y el ondansetrón es un antagonista específico de la serotonina para el receptor 5-HT3 y, rutinariamente, se emplea durante el período postoperatorio para el control de náuseas y vómitos^24^. 

Aunque este estudio no tiene la intención de presentar el ondansetrón como una estrategia farmacológica para el manejo del *delirium*, se destaca que la no utilización de este fármaco durante el período intraoperatorio se asoció con un incremento del 198% (OR: 2,895) en la posibilidad de ocurrencia de *delirium*. Por lo tanto, este hallazgo tiene relevancia por proporcionar subsidios que fomentan la hipótesis relacional de los neurotransmisores desencadenantes del *delirium*, especialmente la serotonina. 

Sin embargo, aunque esta investigación ha evidenciado la asociación de factores predisponentes y precipitantes con el *delirium*, algunas limitaciones deben ser explicitadas, como la no individualización respecto a las dosis de los medicamentos evaluados, así como el escenario de estudio ser un hospital universitario de alta complejidad que no cuenta con atención de emergencia, suscitando la posibilidad de que la población estudiada posea características quirúrgicas diferenciadas, y que los hallazgos puedan no ser generalizables.

Además, se destaca la imposibilidad de obtener algunas variables de estudio para todos los participantes, que fueron tabuladas como datos faltantes debido a la incompletitud de la información en los historiales médicos. Otras no pudieron ser evaluadas por este estudio, como el estado nutricional previo y la clasificación de riesgo quirúrgico según la *American Society of Anesthesiology* (ASA), datos considerados relevantes para la ocurrencia de *delirium*, pero ausentes en los historiales de los participantes.

Se destaca que, aunque existe una correlación entre los factores precipitantes y predisponentes con el *delirium* postoperatorio, se entiende que esta no establece causalidad y que los factores asociados pueden estar relacionados con una causa subyacente aún no esclarecida en la literatura. 

En este caso, se sugiere que la identificación de los factores de riesgo realizada por este estudio contribuya a la orientación científica sobre la elucidación fisiopatológica del *delirium* y también fundamente futuras publicaciones enfocadas en la validación de intervenciones que busquen reducir el riesgo para DPO.

## Conclusión

Se constató que el *delirium* representa un acometimiento frecuente durante el período postoperatorio de adultos críticos. Teniendo en cuenta la terapia intensiva como un ambiente propicio para tal, ya sea debido a la situación de confinamiento subyacente o por la condición orgánica desfavorable de sus pacientes, y teniendo como evento catalizador el procedimiento anestésico-quirúrgico, se postula como estrategia mitigadora la identificación de factores asociados al *delirium*.

Aunque algunos factores predisponentes para el *delirium* están bien establecidos en la literatura, este estudio se diferencia por establecer los factores precipitantes relacionados con el período intraoperatorio, como el tiempo quirúrgico, el uso de sufentanilo, el empleo de transfusión sanguínea, la infusión de agentes anestésicos, así como la no administración de ondansetrón, un antagonista de la serotonina y, por lo tanto, corrobora con la hipótesis relacional de neurotransmisores para el desarrollo de *delirium*.

De este modo, se destaca la actuación del enfermero en las terapias intensivas quirúrgicas tanto para la identificación del *delirium* mediante el uso de instrumentos validados, como para la detección de factores asociados, especialmente en ancianos y pacientes que se sometieron a cirugías prolongadas, los cuales tienen mayores chances de desarrollar esta disfunción. Con esto, se pretende ofrecer insumos que orienten la sistematización de la asistencia de Enfermería perioperatoria, en la medida en que este trabajo agrega complementariedad al diagnóstico de Enfermería denominado confusión aguda.

Se postula que este estudio proporcione una amplia contribución social en la medida en que aborda una entidad nosológica frecuente en las personas mayores en un panorama global de envejecimiento poblacional. Se cree aún en la reducción de los costos socioeconómicos como consecuencia de la mejora de la calidad de los cuidados de salud, así como el aumento de la expectativa de vida y fomento para la implementación de tecnologías de cuidado innovadoras.
